# The methionine salvage pathway-involving ADI1 inhibits hepatoma growth by epigenetically altering genes expression via elevating S-adenosylmethionine

**DOI:** 10.1038/s41419-019-1486-4

**Published:** 2019-03-11

**Authors:** Yu-De Chu, Hsin-Yu Lai, Li-Mei Pai, Ya-Hui Huang, Yang-Hsiang Lin, Kung-Hao Liang, Chau-Ting Yeh

**Affiliations:** 1 Liver Research Center, Chang Gung Memorial Hospital, Taoyuan, Taiwan; 2 Molecular Medicine Research Center, Chang Gung University, Taoyuan, Taiwan; 3 Department of Biochemistry, Chang Gung University, Taoyuan City, Taiwan; 4 Graduate Institute of Biomedical Sciences, College of Medicine, Chang Gung University, Taoyuan City, Taiwan; 5 Medical Research Department, Taipei Veterans General Hospital, Taipei, Taiwan

## Abstract

The 5′-methylthioadenosine (MTA) cycle-participating human acireductone dioxygenase 1 (ADI1) has been implicated as a tumor suppressor in prostate cancer, yet its role remains unclear in hepatocellular carcinoma (HCC). Here, we demonstrated a significant reduction of ADI1, either in protein or mRNA level, in HCC tissues. Additionally, higher ADI1 levels were associated with favorable postoperative recurrence-free survival in HCC patients. By altering ADI1 expression in HCC cells, a negative correlation between ADI1 and cell proliferation was observed. Cell-based and xenograft experiments were performed by using cells overexpressing ADI1 mutants carrying mutations at the metal-binding sites (E94A and H133A, respectively), which selectively disrupted differential catalytic steps, resulting in staying or leaving the MTA cycle. The results showed that the growth suppression effect was mediated by accelerating the MTA cycle. A cDNA microarray analysis followed by verification experiments identified that caveolin-1 (CAV1), a growth-promoting protein in HCC, was markedly decreased upon ADI1 overexpression. Suppression of CAV1 expression was mediated by an increase of *S*-adenosylmethionine (SAMe) level. The methylation status of *CAV1* promoter was significantly altered upon ADI1 overexpression. Finally, a genome-wide methylation analysis revealed that ADI1 overexpression altered promoter methylation profiles in a set of cancer-related genes, including *CAV1* and genes encoding antisense non-coding RNAs, long non-coding RNAs, and microRNAs, resulting in significant changes of their expression levels. In conclusion, ADI1 expression promoted MTA cycle to increase SAMe levels, which altered genome-wide promoter methylation profiles, resulting in altered gene expression and HCC growth suppression.

## Introduction

During the past decades, cancer becomes a leading cause of human death^1^. Among the most common cancer types, hepatocellular carcinoma (HCC) accounts for the third leading cause for cancer-related death^2^. Of all therapeutic modalities, surgical removal of the liver tumorous part remains the most effective treatment^2^. However, only a subset of patients in early cancer stage are qualified for surgical resection^3^. Recently, there are emerging therapies such as transcatheter arterial chemoembolization using new embolizing materials and oral targeted drugs, sorafenib for instance, to treat patients with unresectable HCC^4^. Yet, the responses to these treatments are usually unsatisfactory^4^. Lacking an effective therapeutic strategy justifies the continuous efforts to investigate detail mechanisms of HCC progression to discover new therapeutic targets.

Previously, we identified the human acireductone dioxygenase (ADI1 or also named Sip-L and MTCBP1) as a hepatic factor serving as an enhancer for hepatitis C virus (HCV) cell entry^5–7^. The evolutionarily conserved role of ADI1 has been defined and classified as a member of cupin protein family, which is one of the most functionally diverse superfamilies^8–11^. As an acireductone dioxygenase, ADI1 participates in the methionine salvage pathway or the 5′-methylthioadenosine (MTA) cycle and requires Fe^2+^ metal ion as a cofactor to execute its function in production of 4-methylthio-2-oxobutanate (MTOB), a key step in the pathway^12^. Alternatively, an “off-pathway” proceeds when a Ni^2+^ is employed to replace Fe^2+^ ion in ADI1 enzymatic center and thus produces 3-methyl-thio-propionate^13^.

Besides serving as a key enzyme in MTA cycle, ADI1 has been implied as a potential tumor suppressor in several types of cancers according to its declined level in cancerous tissues^11,14,15^. The mechanisms by which ADI1 functions as a tumor suppressor remain varied and elusive. One proposed model suggested ADI1 represses tumor progression via physically interacting with MT1-MMP, an oncogenic protein, and thus abrogating the induced autophagy progression^11,15^. Another possible explanation was provided by Oram et al.^14^, where they demonstrated that elevated expression of ADI1 in prostate cancer cells was correlated to a higher apoptotic rate for an unknown reason. Intriguingly, increased apoptosis was also observed when directly supplementing MTOB in the growth media of pancreas carcinoma, breast cancer, and HCC cell lines^16^. These findings imply an alternative possibility that the tumor suppressive role of ADI1 is contributed from the enzymatic activity to produce MTOB or downstream metabolites in MTA cycle^11,14^.

Here, we aimed to investigate the role of ADI1 in HCC through clinical correlation, cell-based and xenograft experiments, and genome-wide methylation analysis.

## Results

### Down-regulation of ADI1 in HCC

**Fig. 1:**
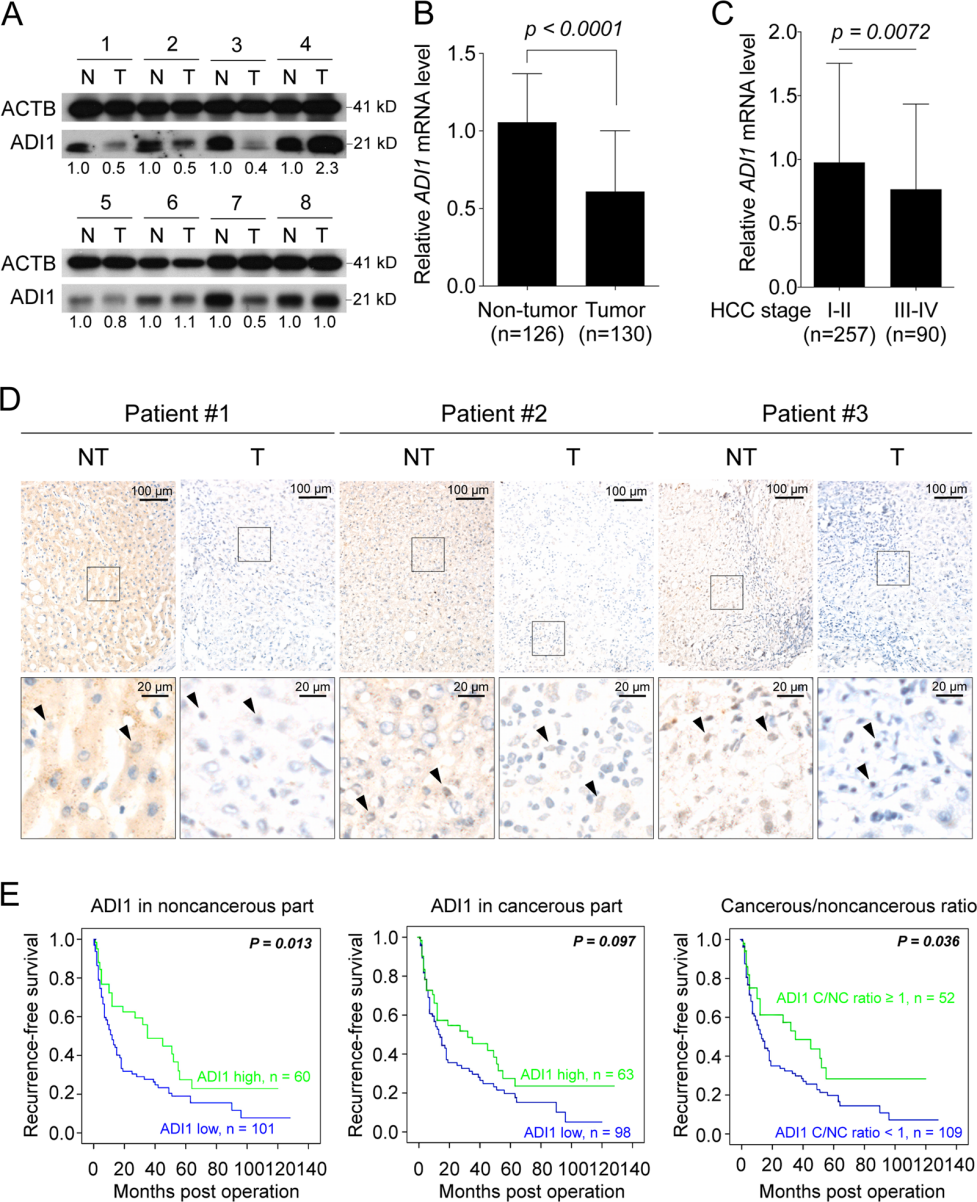
ADI1 was significantly down-regulated in hepatocellular carcinoma (HCC) tissues and its levels were associated with postoperative prognosis in patients with HCC. **a** ADI1 levels in tumorous (T) and non-tumorous (N) parts were analyzed by western blotting. ACTB, beta-actin, was used as a loading control. **b** GSE14520 and **c** TCGA dataset were utilized to acquire the transcript levels of ADI1 for cross-reference. The *p* value was derived by using the unpaired two-tailed Student's *t*-test. The short vertical bars represented standard deviations. **d** IHC was performed to examine the subcellular distribution of ADI1 in tissues derived from HCC patients. Black arrowheads indicate the positive signal of ADI1 in nuclei. The black line represents the scale bar as indicated. **e** The protein levels of ADI1 obtained from 161 patients-derived cancerous and non-cancerous tissues were correlated with recurrence-free survival according to the amount in non-tumorous (left), tumorous (middle), and the ratio of tumorous/non-tumorous (right) tissues

As an attempt to investigate the role of ADI1 in HCC, we performed western blot to assess its levels in cancerous and non-cancerous tissues derived from 161 patients. The baseline clinicopathological information of these patients is listed in Supplementary Table S1. Interestingly, a large proportion of patients exhibited significant reduction of ADI1 in the tumorous parts, compared to those of non-tumorous parts from the same patients (Fig. 1a). To gain supporting evidence, the GSE14520 dataset was employed to assess whether *ADI1* mRNA also down-regulated. Consistently, the *ADI1* transcript significantly reduced in the cancerous part, implying down-regulation of ADI1 might originate from low mRNA expression (Fig. 1b).

If ADI1 plays a tumor suppressive role in HCC, a decreased level of it when tumor progressed to a higher grade should be observed. To examine this view, the TCGA database was used. The objects were assorted with traceable tumor grade to stage I–II or III–IV. Analysis of these two groups revealed that, indeed, the *ADI1* mRNA level was significantly reduced along with the progression of HCC to a higher grade (Fig. 1c).

Previously, it has been demonstrated that ADI1 both localized in the cytosolic and nuclear compartments^7,14^. However, the subcellular localizations of ADI1 in HCC tissues remain unclear. To address this issue, Immunohistochemistry (IHC) analysis was performed. It was found that ADI1 decreased in the tumorous parts (Fig. 1d), especially in cytoplasm, when compared to that in non-tumorous parts, consistent with the observation in western blot analysis (Fig. 1a). Additionally, it was found that the nuclear ADI1 was presented in the cancerous tissues in 72/161 (44.7%) and in the non-cancerous tissues in 10/161 (6.2%) patients, respectively (*p* < 0.001). Yet, neither the nuclear ADI1 in the cancerous parts nor the nuclear ADI1 in the non-cancerous parts was associated with postoperative HCC recurrence (recurrence-free survivals, *p* = 0.072 and 0.664, respectively).

### ADI1 level positively associated with postoperative recurrence-free survival in patients with HCC

Subsequently, we sought to determine the clinical relevance of ADI1 level in term of postoperative prognosis in HCC patients. Kaplan–Meyer analysis was employed to compare between the subgroups with high and low ADI1 levels (using the mean of ADI1 protein levels as cutoff, after normalized with that of primary hepatocytes) (Supplementary Fig. S1A). The recurrence-free survival was significantly associated with ADI1 protein expression in the non-tumorous (*p* = 0.013) (Fig. 1e, left panel) but not the tumorous parts (*p* = 0.097) (Fig. 1e, middle panel). However, patients with higher ADI1 levels in the tumorous parts still had a better prognosis, albeit not statistically significant. This could be caused by variations of the ADI1 levels in the corresponding non-cancerous parts. Therefore, to decipher the growth regulatory role of ADI1, it was more precise to examine the survival curves stratified by the T/N ratio (Fig. 1e, right panel). The result showed that a higher cancerous/non-cancerous ADI1 ratio (in each patient) was associated with a better prognosis.

Consistently, we found a better prognosis in 144 patients with higher *ADI1* mRNA levels, while a poorer prognosis in 218 patients with lower *ADI1* mRNA levels (*p* = 0.0375), retrieved from the TCGA database (Supplementary Fig. S1B).

### Alteration of ADI1 expression affected hepatoma cell proliferation

**Fig. 2:**
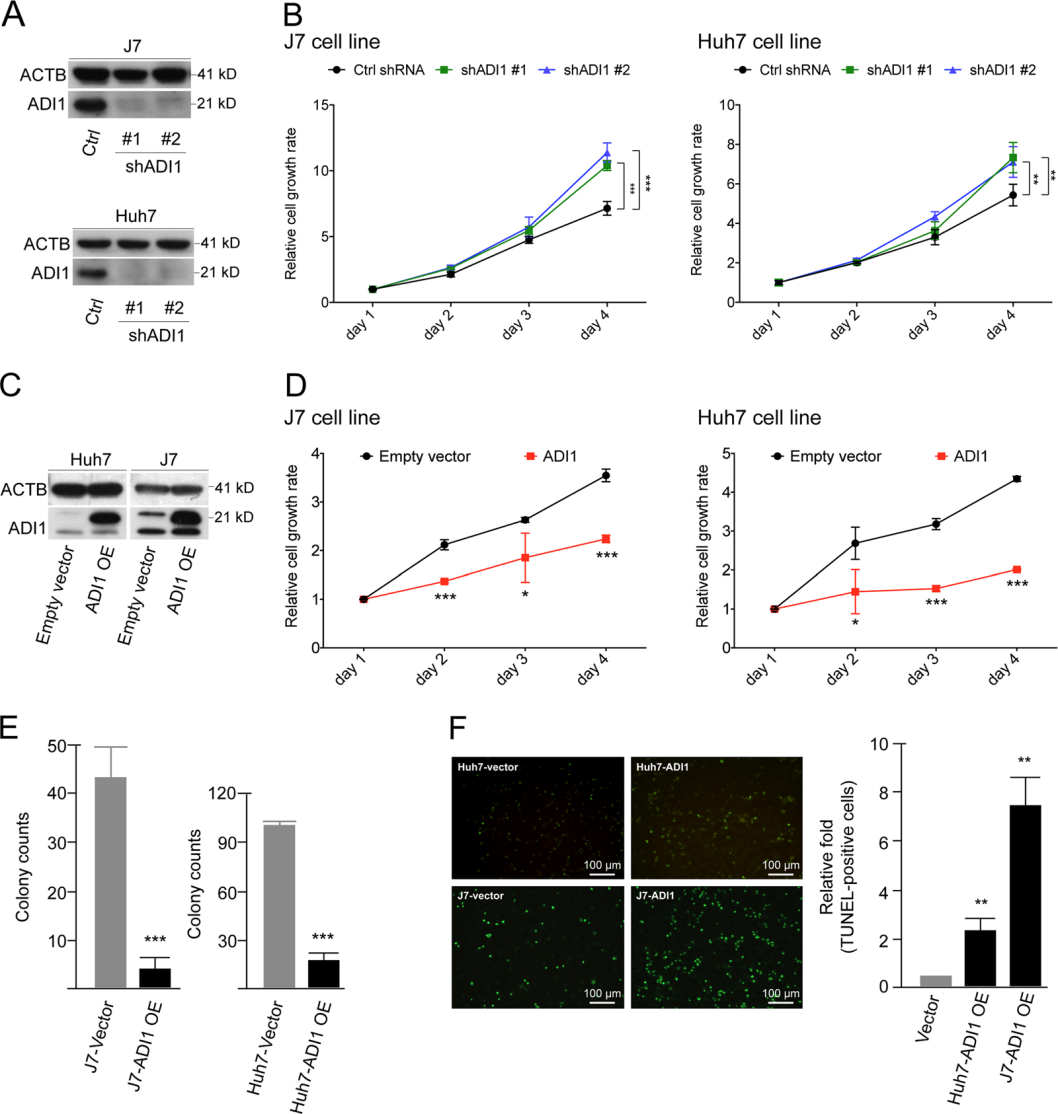
ADI1 suppressed hepatocellular carcinoma (HCC) cell proliferation. **a** A representative western blot analysis of samples derived from stably ADI1-knockdown in J7 (upper) and Huh7 (lower) cells. Two targets (#1 and #2) for shADI1-mediated knockdown were performed. **b** The proliferation rates were determined by measuring the florescence of metabolite derived from alarmar blue in J7 (left) or Huh7 (right) cells. Black, mock control (Ctrl); green, shADI1#1 knockdown; blue, shADI1#2 knockdown. **c** Western blots of ADI1 overexpressing (OE) in Huh7 or J7 cells. **d** Proliferation rates of mock (empty vector, black) or ADI1-overexpressing cells (red) on days 1–4 post seeded to a 96-well plate. **e** Colony formation assay of ADI1 overexpressing (OE) in J7 (left) and Huh7 (right) cells. **f** The representative image of apoptosis status in Huh7 and J7 cells with or without ADI1 overexpression (OE). The *p* values were derived by using unpaired two-tailed Student's *t*-test. **p < 0.01. The white line represented the scale bar as indicated. The short vertical bars represented standard deviations

To investigate the issue whether ADI1 served as a tumor suppressor in HCC, proliferation rate was assessed in cells with altered ADI1 expression. To mimic the situation of low ADI1 expression in tumorous tissues, the lentivirus-mediated down-regulation was conducted by using two shRNA clones (Fig. 2a). Notably, we found depletion of ADI1 significantly enhanced cell proliferation (Fig. 2b). Conversely, overexpression of ADI1 led to opposite effects (Fig. 2c–e).

To examine whether the reduced doubling time of ADI1-overexpressing cells was due to the increased apoptosis rate as reported previously^14^, we performed terminal deoxynucleotidyl transferase dUTP nick end labeling (TUNEL) assay to determine the status of cell apoptosis. Consistently, we found the ratio of apoptotic cells significantly elevated when cells overexpressing ADI1 (Fig. 2f).

### The growth suppression effect of ADI1 was mediated through MTA cycle

**Fig. 3:**
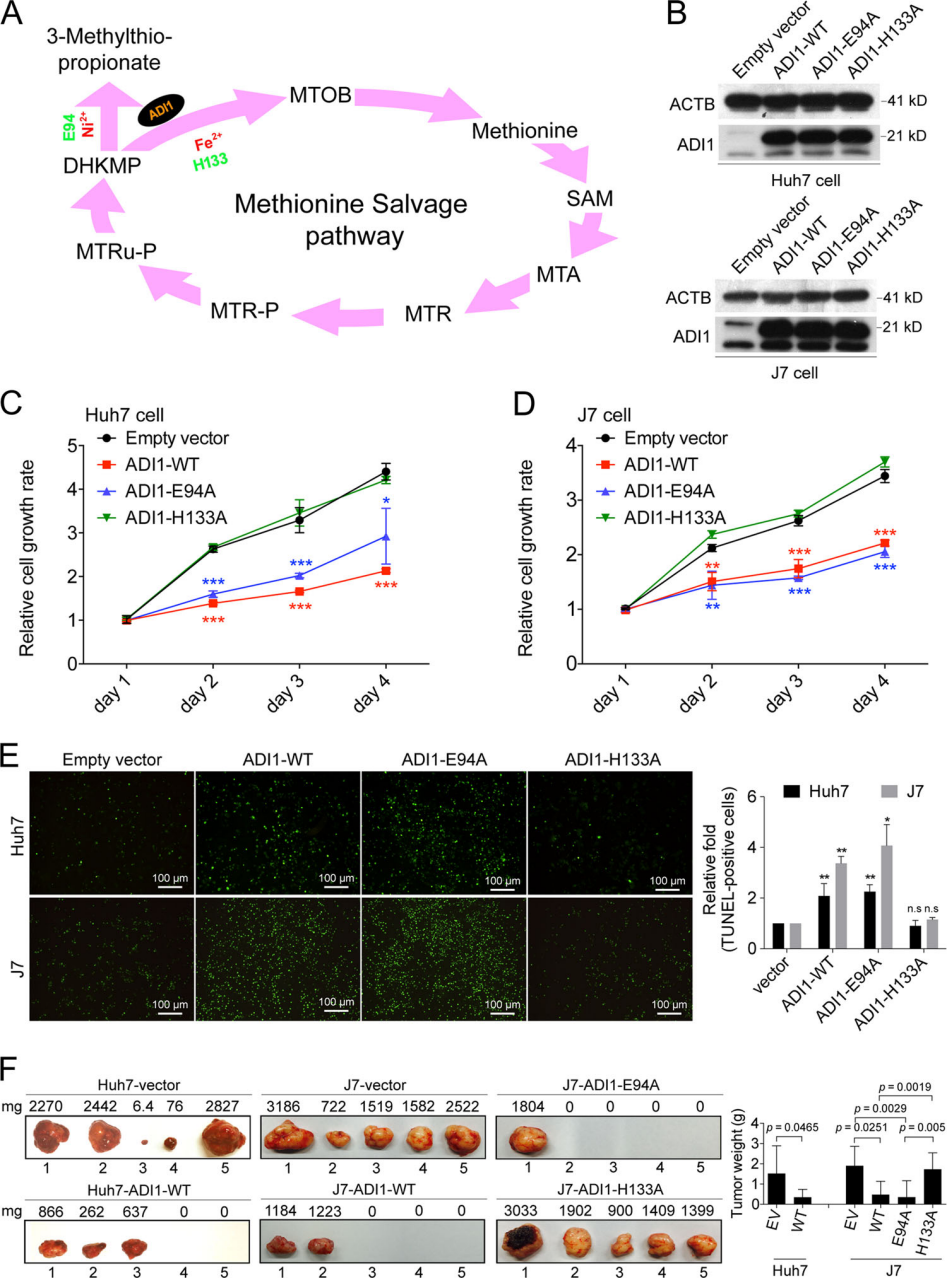
Growth inhibition effect mediated by ADI1 is through MTA cycle. **a** Schematic representation of steps in MTA cycle. MTOB, 2-keto-4-methylthiobutyrate; SAMe, *S*-adenosylmethionine; MTA, 5-methyl-thioadenosine; MTR, 5-methyl-thioribose; MTR-P, 5-methyl-thioribose-1-P; MTRu-P, 5-methyl-thioribulose-1-P; DHKMP, 1,2-dihydroxy-3-keto-5-methylthiopentene. **b**, **c** Western blot analysis of mutated ADI1 (ADI-H133A and ADI-E94A) stably expressed in Huh7 and J7 cells. **d**, **e** The proliferation rates obtained by measuring the florescence of metabolite derived from alamar blue in Huh7 and J7 cells. The *p* value was derived by using unpaired two-tailed Student's *t*-test. **p < 0.01, ***p < 0.001. **f** Xenograft tumors after 4 weeks of subcutaneous injection into nude mice. Huh7 stably expressing mock control or ADI1-WT were shown (left); J7 cells stably expressing mock control, ADI1-WT, ADI1-E94A or ADI1-H133A, and mock control were shown (right). The *p* values were derived by using paired one-tailed Student's *t*-test. The short vertical bars represented standard deviations

The metal-binding ADI1 is well-known for its enzymatic role in MTA cycle^12^. Distinction between the on- or off-pathway mediated by ADI1 can be achieved by different ions associated with the enzyme^13^. Key residues for ion binding were identified and validated, such as the E94 for Ni^2+^ and H133 for Fe^2+^ (Fig. 3a)^13,14^. We then attempted to determine whether metal-binding motif was required for ADI1-mediated effect on cell proliferation. As such, Huh7 and J7 cells transfected with ADI1 carrying either wild type or mutant, E94A or H133A mutation (Fig. 3b), were used for examining the effect of growth inhibition. Interestingly, a markedly decrease of cell proliferation was found in cells expressing ADI1 with E94A mutation, but not in the one with mutation at H133A (Fig. 3c, d).

To examine whether these mutants also altered cell apoptosis as observed in cells carrying wild-type ADI1 (Fig. 2f), additional TUNEL assays were performed. Consistent with the effects of these mutants in cell growth inhibition, it was found that cells expressing ADI1-E94A mutant had an increased percentage of apoptotic cells similar to those expressing wild-type ADI1, but the increment was not found in those expressing H133A (Fig. 3e). The results suggested that the growth suppression effects caused by ADI1 and mutants were partly due to their abilities to induce cell apoptosis in HCC cells.

To further confirm the in vitro findings, we performed xenograft experiments. Consistent with our cell-based results, it was found that the tumor volume of xenografts expressing wild-type ADI1 was significantly smaller than that in the control group. Similarly, the xenografts expressing the ADI1-E94A also manifested a reduced capability of tumor growth in nude mice. However, xenografts expressing ADI1-H133A did not display a growth suppression effect (Fig. 3f). These data suggested that the growth suppression effect of ADI1 was mediated through the MTA cycle.

### ADI1 inhibited Caveolin-1 (CAV1) expression through MTA cycle to modulate hepatoma cells growth

**Fig. 4:**
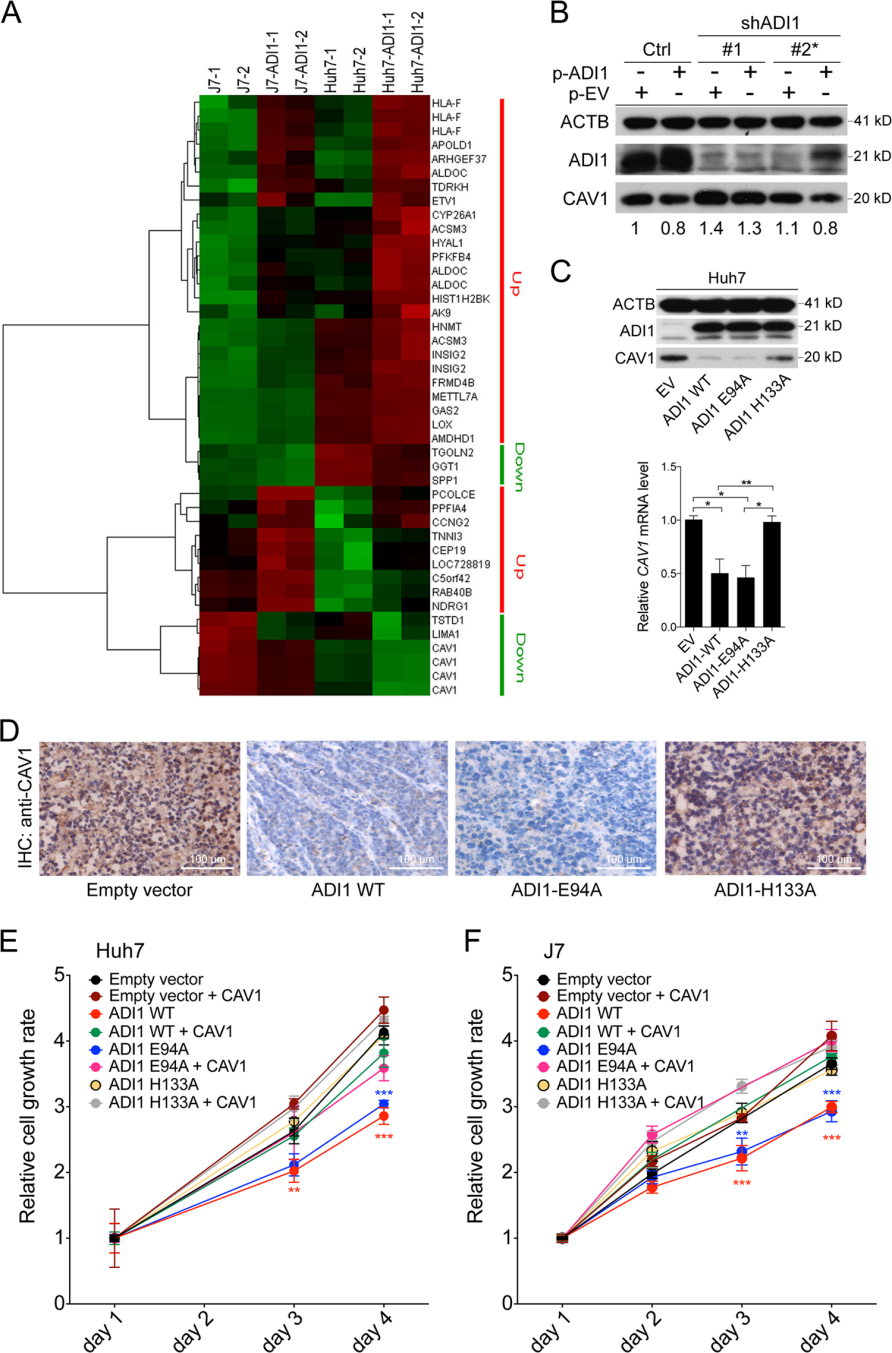
CAV1 acted as a downstream target of MTA cycle-participating ADI1 in cell proliferation control. **a** The heat map represented the relative levels of transcripts under overexpression of ADI1 in J7 and Huh7 cells. Two independent cell clones expressing ADI1 (−1 and −2) were subjected into cDNA array analysis. **b** Western blot analysis for ADI1 and CAV1 expression levels under shADI1#1 and #2 differential target knockdown, with or without extraneous ADI1 complementary expression. p-ADI1, ADI1-expressing plasmid; EV, expressing vector only; ACTB, beta-actin, was used as a loading control. **c** Effect of ADI1 mutants on *CAV1* expression in Huh7 cells. Upper panel, western blot; lower panel, relative amounts of *CAV1* mRNA obtained by RT-qPCR. ***p < 0.001. **d** IHC analysis of CAV1 expression in tissues derived from xenografts (see Fig. 3F). The white line represents the scale bar as indicated. **e**, **f** The cell proliferation rate was assessed by detecting the florescence of metabolite derived from alarmar blue in Huh7 and J7 cells with indicated treatments. The *p* value was derived by using unpaired two-tailed Student *t*-test. **p < 0.01, ***p < 0.001. The short vertical bars represented standard deviations

Subsequently, to search for downstream effectors, we performed cDNA microarray analysis to identify genes with altered expression levels upon ADI1 overexpression. A subset of dysregulated gene was identified (Fig. 4a). Among them, *Caveolin-1 (CAV1)* was decreased most dramatically, more than two-folds (Fig. 4a). The caveolae component, CAV1, has been considered as an enhancer of hepatocarcinogenesis due to its ability to promote cell proliferation and invasion through several well-characterized HCC-related signaling pathways^17^. To validate the impact of ADI1 on CAV1 expression, western blot analysis and reverse transcriptase quantitative PCR (RT-qPCR) were performed in ADI1 stably expressing cells. As shown in Supplementary Fig. S2A, B, we found the protein and mRNA levels of Caveolin-1 remarkably reduced in ADI1-overexpressing cells. Conversely, knockdown of ADI1 significantly induced CAV1 protein and mRNA expression (Supplementary Fig. S2C, D). To more comprehensively examine the relationship between ADI1 and CAV1, we performed a complementation assay. In this experiment, two independent shRNA clones, #1 targeted exon yet #2 recognized the 3′UTR sequences of *ADI1* mRNA, were employed. As a result, we found that #1 effectively diminished ADI1 expression despite of replenishing it extraneously by transfecting ADI-expressing plasmids in Huh7 and J7 cells (Fig. 4b, and Supplementary Fig. S2E). As anticipated, it was found that the shRNA #2 also effectively reducing intrinsic ADI1 expression. However, it was unable to reduce the level of extraneous ADI1 since no 3′UTR was present for targeting (Fig. 4b, Supplementary Fig. S2E). Under any condition, CAV1 levels were always negatively regulated by ADI1 (Fig. 4b), indicating that the regulation was not caused by an off-target effect.

According to the results in Fig. 3, we were then curious whether down-regulation of CAV1 was an event downstream of the MTA cycle. Interestingly, we found that only the “on-pathway” ADI1 (wild type or ADI1-E94A) significantly reduced *CAV1* expression but not the “off-pathway” ADI1-H133A (Fig. 4c, Supplementary Fig. S2F). The IHC analysis of tissues derived from xenografts in Fig. 3f was also in agreement with such notion (Fig. 4d), suggesting the ADI1-mediated suppression of CAV1 was MTA cycle-dependent.

To further examine whether CAV1 was one of the potent downstream effectors in ADI1-mediated repression of cell proliferation, the rescue experiment was performed. As shown in Fig. 4e, f, it was found that the suppressed cell growth could be partially restored by simultaneous expression of CAV1 in HCC cells overexpressed with either wild type or E94A mutated ADI1 (Supplementary Fig. S2F). This observation strongly suggested that the ADI1–CAV1 regulatory axis was required for ADI1-mediated cell growth control.

### ADI1 negatively correlated with CAV1 expression in HCC patients

Next, we investigated whether the negative association between ADI1 and CAV1 levels could be observed in human HCCs. IHC was performed in cancerous and non-cancerous liver tissues. As shown in Supplementary Fig. S3A, B, it was found that ADI1 was significantly reduced in tumorous parts, compared to that in adjacent non-tumorous tissues, while a marked reciprocal expression pattern of CAV1 was found in the tumorous and non-tumorous liver tissues, indicating a negative association between these two proteins in human HCCs.

Furthermore, *CAV1* mRNA was increased in higher-grade HCC tissues compared with lower-grade group in TCGA dataset (Supplementary Fig. S3C). Also, an elevated amount of *CAV1* transcript was correlated with poorer clinical outcome, while a lower level predicted better prognosis in HCC patients (Supplementary Fig. S3D). Similar results were observed in GSE14250 dataset (Supplementary Fig. S3E). Further stratification of these patients by *ADI1* level revealed that in both cancerous and non-cancerous tissues, a negative association between *ADI1* and *CAV1* levels was present (Supplementary Fig. S3F).

### ADI1-mediated *S*-adenosylmethionine elevation altered methylation status in *CAV1* promoter

**Fig. 5:**
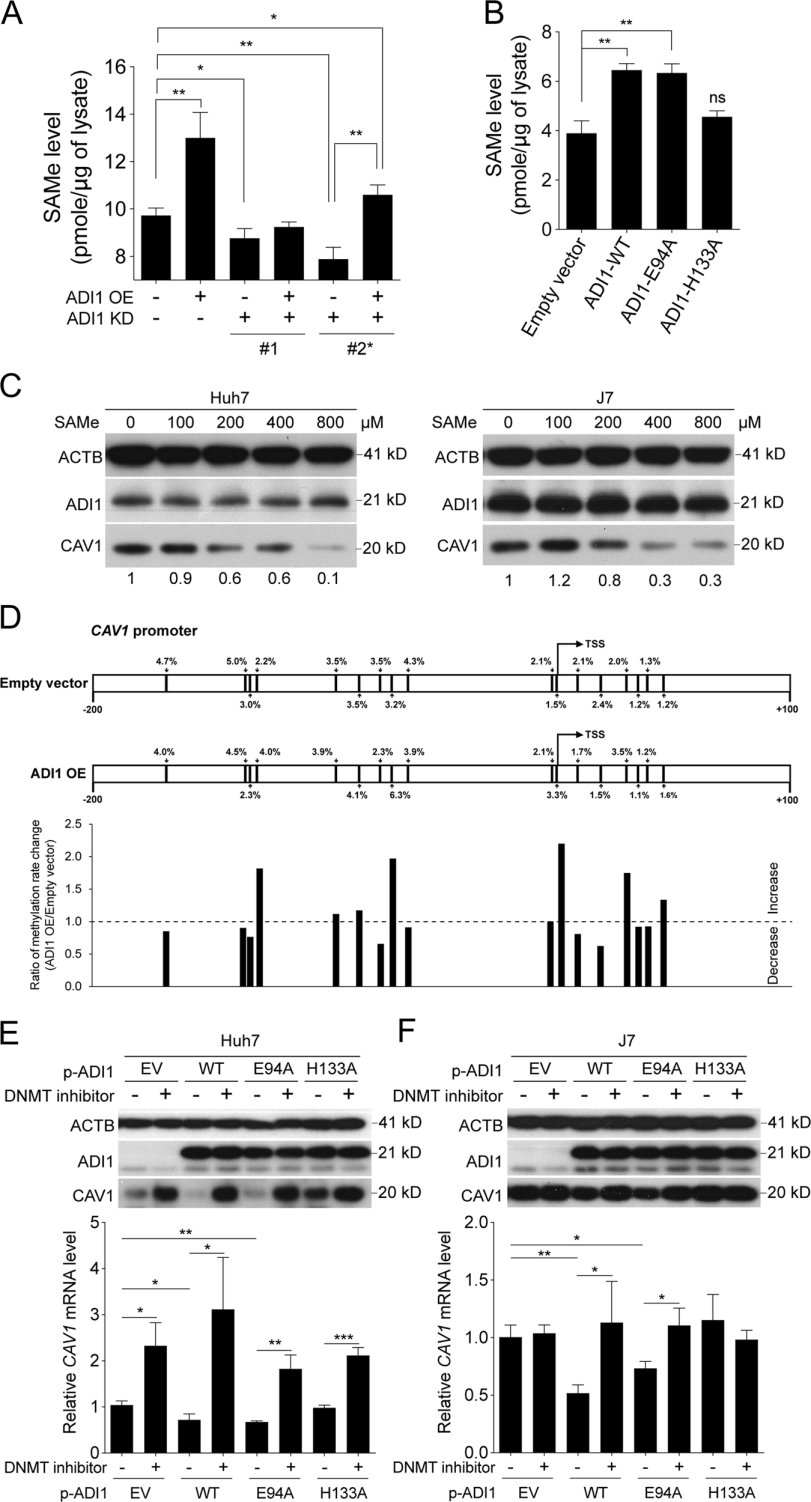
ADI1 increased SAMe levels and suppressed CAV1 expression by altering promoter methylation status. **a** Quantification of SAMe levels in Huh7 cells with knockdown (KD) or complementary extraneous expression (OE) of ADI1. **b** Quantification of SAMe levels in Huh7 cells transiently expressing wild type (WT) or mutant ADI1s. *P* values were calculated by unpaired two-tailed Student's *t*-test. *p < 0.05, **p < 0.01, ***p < 0.001. **c** Western blot analysis of lysates from Huh7 or J7 cells pre-treated with SAMe in indicated concentrations. **d** The bisulfite converted *CAV1* promoter region was analyzed by next-generation sequencing in J7 cells with or without ADI1 overexpression (OE). The percentage of methylated CpG islands were labeled. TSS, transcription start site. Western blot and RT-qPCR analysis of samples from empty vector transfected or ADI1-expressing **e** Huh7 or **f** J7 cells pre-treated with or without DNMT inhibitor. The short vertical bars represented standard deviations

Next, to investigate the underlying mechanism employed by ADI1, we asked whether change of metabolite concentrations in the MTA cycle affected CAV1 expression. Accordingly, we assessed the level of the final product in pathway, *S*-adenosylmethionine (SAMe), upon altered expression of ADI1. As a consequence, it was found that SAMe concentrations were positively associated with the levels of ADI1 (Supplementary Fig. S4A, B). More systemically examination by employing the samples from complementation assay also exhibited strong correlation between ADI1 and SAMe levels (Fig. 5a). Furthermore, mutation at H133 disrupted the effectiveness of ADI1 in enhancing SAMe synthesis (Fig. 5b).

It is uncertain whether elevation of SAMe concentration had an impact on *CAV1* expression. To examine this point, we collected cells and analyzed the CAV1 level following SAMe treatment in various concentrations for 72 h. As shown in Fig. 5c, it was found that the CAV1 protein level decreased in an SAMe dosage-dependent manner. Additionally, it would be interesting to understand if the levels of ADI1 could change in cells treated with distinct SAMe dosages. The results showed that no significant difference of ADI1 levels was found under the indicated SAMe concentrations, suggesting that there was no prominent feedback loop for SAMe to modulate ADI1 expression (Fig. 5c).

Subsequently, owing to the role of SAMe in cellular methylation process, we aimed to investigate whether methylation status of *CAV1* promoter was altered when overexpressing ADI1 in HCC cell lines. To achieve this, we isolated genomic DNAs and performed bisulfite conversion. It was then served as a template to amplify the promoter region of *CAV1* as previously described^18^. After next-generation sequencing analysis, we found the ratio of several CpG islands, especially the one just one base-pair prior to the transcription start site (TSS), significantly elevated upon ADI1 overexpression either in J7 (Fig. 5d) or Huh7 cells (Supplementary Fig. S4C).

To determine whether the CpG island methylation in the promoter region was important for regulation of CAV1 expression, the inhibitor of DNA methyl transferase (DNMT) was employed in cells with or without ADI1 overexpression. As anticipated, expression of CAV1 was significantly elevated under supplementing DNMT inhibitor, in cells with either wild type or E94A mutated ADI1 overexpression (Fig. 5e, f), confirming the hypothesis that ADI1 increased SAMe and therefore enhanced *CAV1* promoter methylation to suppress its expression.

### ADI1 modulated promoter methylation status of a subset of genes in HCC cells

**Fig. 6:**
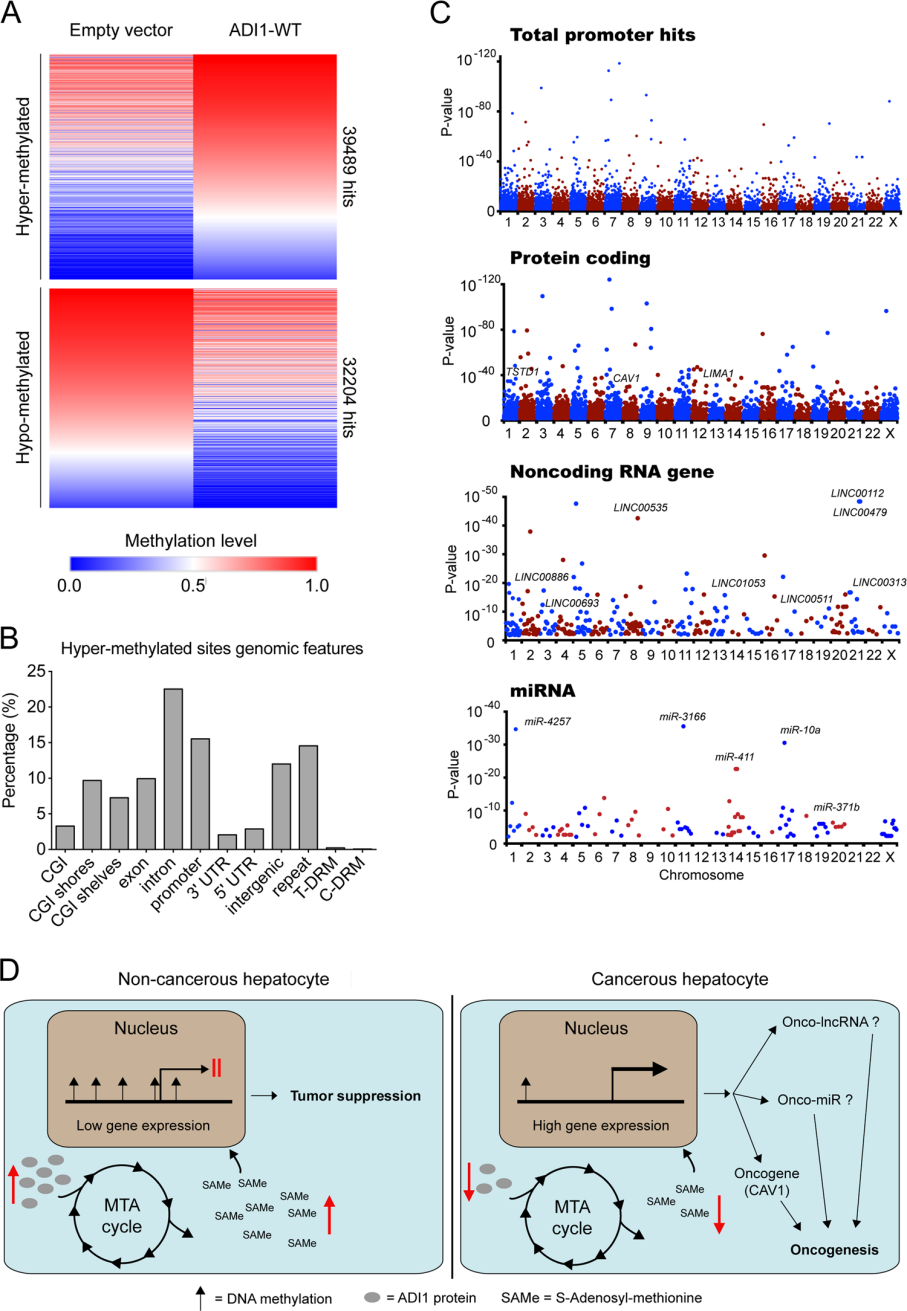
Promoters of a set of genes were hyper-methylated in ADI1-overexpressing cells. **a** Heat map representation of DNA methylation levels in J7 cell with or without ADI1 overexpression. **b** The genomic features of hyper-methylated sites in ADI1-overexpressing cells. **c** The distributions of significant hypermethylation in total promoters (upper), promoters for protein coding genes (upper middle), promoters for non-coding RNA genes (lower middle), and promoters for miRNA genes (lower). Horizontal axis, chromosome numbers; vertical axis, *p* value. **d** The proposed growth regulatory mechanism obtained from this study. In non-cancerous hepatocytes, ADI (gray oval) levels were high, promoting MTA cycle to generate a large amount of SAMe, which in term modulated genome-wide promoter methylation to achieve a gene expression pattern for tumor suppression. In cancerous hepatocytes, ADI1 was reduced, leading to reduction of SAMe concentrations and thus alterations of genome methylation pattern. As a result, several oncogenes (such as *CAV1*), lncRNA, and miRNA were activated to promote cancer cell growth

To explore the impact of ADI1 on DNA methylation, we performed methyl-seq analysis and compared the whole-genome methylation status between control and ADI1-overexpressing cells. As a consequence, we identified a large number of sites, which were hyper- or hypo-methylated when overexpressing ADI1 (Fig. 6a). Further examination of the genomic features of these sites revealed nearly 15% of identified sites were located in the gene promoter regions (Fig. 6b). Among these genes, most of them were protein coding genes, including those with declined mRNA observed in cDNA microarray analysis (Fig. 4a), such as *TSTD1*, *LIMA1*, and *CAV1* (Fig. 6c).

Additionally, the promoter methylation status of some non-coding RNA (ncRNA) genes, including antisense RNAs, long intergenic non-coding RNAs (lincRNAs), and miRNA, were also altered (Fig. 6c). To validate whether ADI1-mediated hypermethylation of these non-coding RNA gene promoter affected their expression, we assessed the expression levels of a panel of genes, which were presumably expressed in hepatic cells, by performing quantitative real-time PCR. Interestingly, we found the expression of some of ncRNA genes actually declined in cells overexpressing ADI1 while increased upon depletion of it (Supplementary Figs. S5–7). By literature searching, we further discovered that some of them were implied as oncogenes in numerous types of cancers, such as TP73-AS1 in HCC^19^ (Supplementary Fig. S5), LINC00511 in non-small cell lung cancer^20^ (Supplementary Fig. S6) as well as miR-371 in HCC and pancreatic cancer^21,22^ (Supplementary Fig. S7), supporting our hypothesis that the expression of genes with hyper-methylated promoter might affect gene expression.

Taken together, we demonstrated that ADI1 served as a tumor suppressor in HCC by modulating productivity of SAMe in MTA cycle. Moreover, a higher amount of SAMe might therefore repress expression of a subset of genes by hypermethylation of their promoters (Fig. 6D).

## Discussion

The human ADI1 has been implicated as a potential tumor suppressor in numerous types of cancers^11,14,15,23^. Here, we demonstrated that ADI1 was significantly reduced, either protein or mRNA level, in HCC. Its expression levels positively correlated with postoperative prognosis of HCC (Fig. 1). These results supported that ADI1 also played a tumor suppressive role in HCC. Moreover, ADI1 expression suppressed HCC cell proliferation (Fig. 2). This could be explained by alteration of apoptosis rate in immortalized hepatic cells when the ADI1 level increased, similar to what was found in prostate cancer (Fig. 2f)^14^. It is appealing to assume that the ability of ADI1 to induce hepatoma cell apoptosis and thus inhibit cell proliferation could be consequences of increasing productivity of MTOB or more downstream metabolites, SAMe for instance, in MTA cycle, as some studies have proposed such possibility^16,24,25^.

Activation of ADI1 enzymatic activity requires metal ion association within the enzymatic center, Fe^2+^ and Ni^2+^ for the on- and off-pathway, respectively (Fig. 3a)^13^. The specific amino acid residues responsible for these ions binding were critically identified in previously reports, the E94 and H133, for Ni^2+^ and Fe^2+^, respectively^9,12,13^. Examination of the role of MTA cycle in cancer cells reproduction by mutating these critical residues in ADI1 revealed that the “on-pathway” implemented by H133 participated in the repression of HCC growth (Fig. 3). It remains disputable to date in the literature whether MTA cycle affects cancer progression^24,26–29^. Our results demonstrated that similar to the findings made in prostate cancer, wild-type ADI1 functions in modulating cell apoptosis and growth^14^. However, mutation in H133 or E94 site did not affect ADI1 effects on apoptosis or cell proliferation in LNCaP cells (a prostate cancer cell line), which was quite different from our findings in HCC cell lines, as H133A mutation abolished both functions of ADI1 in cell growth inhibition and apoptosis enhancement (Fig. 3). It is possible that the tumor suppression effect of ADI1 in HCC is mainly manifested through the MTA cycle-mediated increment of SAMe, but might not be so in prostate cancer, because the rate-limiting step for synthesis of SAMe depends on the level of methionine adenosyltransferase in cells. There are two methionine adenosyltransferase isozyme-encoding genes in human chromosome, *MAT1A* and *MAT2A*. It is well-known that the gene product of *MAT1A* is a liver-specific protein and is required for SAMe synthesis. Lowering the ratio of the gene products MAT1A/MAT2A has been shown to correlate with a sharp decrease of SAMe level in hepatocytes as well as in HCC cells (for a review, see ref. ^30^). Further, higher levels of SAMe have been found to induce apoptosis. Taken together, although we could not exclude the possibility that there might be other MTA-cycle-independent pathways involved, the present study strongly suggested that the MTA cycle and/or its downstream metabolite played a major role in ADI1-mediated tumor suppressive effect.

On the other hand, there were a borderline association between postoperative HCC and nuclear ADI1 (*p* = 0.072) and a higher percentage of nuclear ADI1 in the cancerous tissues (*p* < 0.001). Further investigation should be conducted to clarify the role of nuclear ADI1 in HCC.

During the past decades, a number of preclinical studies have demonstrated the possibility of using SAMe for anti-HCC purpose, such as in vitro and in vivo apoptosis enhancement, growth, and angiogenesis inhibition of HCC^25,31–34^. Furthermore, for clinical usage of SAMe, lines of evidences have demonstrated significantly improvement of hepatic functions in patients with chronic conditions such as cholestasis or alcoholic liver disease^35,36^. Similarly, reduction of alfa-fetoprotein and exhibition of protective capability were also observed under treatment of SAMe in patients with HCV-related cirrhosis and HBV-associated HCC, respectively^37,38^.

SAMe has been well-known for its ability to serve as a methyl donor in methyltransferation and induces methylation on targets^39^. Characterization of substrates for transferring the methyl-group revealed that not merely DNA, but protein and RNA were also capable of methylation^40^. In such case, despite we have observed an elevation of methylation on CpG island of *CAV1* promoter and in promoters of a set of genes from cells overexpressing ADI1, in which SAMe synthesis was enhanced (Figs. 5 and 6), we still cannot exclude the possibility that the mRNA or protein derived from other genes were also methylated and thus affected cell growth.

In summary, our present study has unraveled the role of ADI1 and MTA cycle in suppression of HCC cell proliferation. The level of ADI1 was positively associated with SAMe production and thus impacted on promoter methylation status of a set of genes including the oncogene *CAV1*. The expression levels of these genes were then altered, leading to cell growth inhibition.

## Materials and methods

### Patients

Three different cohorts of HCC patients were analyzed. Firstly, 161 paired tumorous and non-tumorous liver tissues used in this study for protein level analysis by western blotting or IHC staining were obtained from Tissue Bank, Chang Gung Memorial Hospital under the permission of the institutional review board, Chang Gung Memorial Hospital, Linkou, Taiwan. Secondly, the online available cDNA microarray dataset, GSE14520, which included the data from 130 tumorous and 126 non-tumorous RNA samples, was used to compare the RNA levels of interested genes. Thirdly, transcriptomic results were also retrieved from the TCGA database (https://genome-cancer.ucsc.edu/), which included 347 RNA-sequencing data that derived from defined tumor stages of HCC patients.

### IHC staining

The IHC staining was executed as described previously^7^. The retrieved surgical resected tumorous and adjacent non-tumorous tissues from our tissue bank were used for IHC staining. The IHC analysis was conducted by using the DAB peroxidase substrate kit (Vector Lab, SK-4100) according to the protocol provided by the manufacturer. To analyze the distribution and expression level of ADI1, a homemade rabbit polyclonal antibody was used. The generation of rabbit polyclonal anti-ADI1 antibody was described in our previously study^6^. A mouse monoclonal anti-CAV1 antibody (Proteintech, 66067-1-lg) was used for staining of Caveolin-1. The dilution of these two antibodies used in IHC was 1:200.

### Lysate preparation and western blot

Protein extraction and western blot analysis were conducted as described previously^41^. The same amount of an aliquot of commercialized primary hepatocyte cell lysate was loaded into each gel as an additional batch-to-batch control and a quantification-referencing indicator (Supplementary Fig. S1A). Antibodies used for detection of ADI1 and Caveolin-1 in western blotting were the same as described in IHC staining in a dilution of 1:2000 and 1:5000, respectively. For detection of ATCB, a rabbit polyclonal antibody (Proteintech, 20536-1-AP) was used with 5000-fold diluted.

### Lentivirus-mediated down-regulation of ADI1

The lentivirus carrying ADI1 shRNA clones were purchased from Academia Sinica RNAi core facility, Taiwan. The lentivirus transduction was conducted as described previously^42^. The clone IDs of ADI1 shRNAs used in this study were TRCN0000290230 for #1 and TRCN0000296534 for #2. The target sequence of these two clones were 5′-GAACTACTCCTGGATGGACAT-3′ and 5′-ACTAACACGTGCCTCGTAAAG-3′ for #1 and #2, respectively. For complementary experiments, the cells used for transient transfection of empty vector or ADI1-expressing plasmid were puromycin selected (3–7 μg/mL) and shRNAs stably expressed.

### Cell culture

The cells used in this study, J7 and Huh7, were cultured in Dulbecco’s modified Eagle's medium in a standard culture condition with 37° and 5% CO_2_ humidified incubator. For stable expression of interested genes or shRNAs, the antibiotic selection was utilized. The cells stably expressing ADI1 were maintained under the presence of 1 mg/mL of G418. The stably expressed ADI1 shRNA were selected by puromycin at the final concentration of 3–7 μg/mL.

### Cell proliferation assay

The cell proliferation rate was assessed as previously described with minor modifications^43^. Briefly, the alamar blue was used to replace Thiazolyl Blue Tetrazolium Bromide (MTT) for the lower cytotoxicity and simplicity.

### Plasmid constructs for ADI1 and CAV1 expression

The DNA oligos listed below were used for ADI1 and CAV1 open reading frame amplification. The underlined sequences indicated the recognition sites for *Hin*dIII and *Eco*RI in primer pair for ADI1, while for *Eco*RI and *Not*I in that for CAV1. Amplicons of wild type or mutant ADI1 and CAV1 were then inserted into pcDNA3.1/V5-HisB mammalian expression vector (Invitrogen, V81020) by using specific restriction sites. All the constructs used in this study were sequence verified by an automatic sequencer.

ADI1 Forward: 5′-AGTTAAGCTTATGGTGCAGGCCTGGTATAT-3′

ADI1 Reverse: 5′-TGCAGAATTCTTAGGCGGTCTGTGCCAGAA-3′

CAV1 Forward: 5′-ATCGGAATTCATGTCTGGGGGCAAATACGT-3′

CAV1 Reverse: 5′-AATTGCGGCCGCCTATATTTCTTTCTGCAAGTTGA-3′

### Colony formation assay

To assess the capability of cell reproduction, the colony formation assay was conducted. The procedure was executed as described in our previous study^42^.

### TUNEL assay

The cells stably expressing control vector or ADI1 were seeded to a 24-well plate with coverslip in the bottom. After 48–72 h, the apoptosis status was determined by TUNEL assay as described previously^42^.

### Xenograft model

The xenograft model was executed under the approval of Chang Gung Institutional Animal Care and Use Committee. Three-week-old BALB/c nude mice purchased from the National Laboratory Animal Center (Taipei, Taiwan) were used. The procedure was described previously^42^.

### cDNA microarray

The cDNA microarray was used for detection of genes mRNA levels and was conducted as previously described^44^.

### RNA isolation and quantitative RT-PCR

The RNA extraction was performed as previously described^45^. The ToolScript MMLV RTase (TOOLS, TGERA04) was utilized for reverse transcription. Prior to RNA subjected into reverse transcription, the DNase digestion was conducted by using Turbo DNase I (Ambion, AM1907). Ten micrograms of total RNA was used for each digestion experiment. The detail procedures were accordance with the standard protocol provided by the manufacturers. Subsequently, 5 μg of DNA digested RNAs were subjected into RT reaction by using ToolScript MMLV RTase (TOOLS, TGERA04). For each 20 μL reaction, 5 μL of 50-fold diluted cDNAs were utilized in RT-qPCR analysis. For quantitation of miRNAs, the tailing reverse transcription was employed as previously described^41^. The Bio-rad CFX96 real time system was used for detection of interested genes. The DNA oligos and primer pairs used for examining gene expression in this study are listed in Supplementary Table S2.

### SAMe detection

For assessment of SAMe levels, the Bridge-It® *S*-Adenosyl Methionine (SAM) Fluorescence Assay Kit was used according to the standard procedures provided by the manufacturer. In Fig. 5A, the cells stably expressing scramble shRNA or ADI1 shRNA were used for transient expression of empty vector or ADI1-overexpressing plasmid. In Fig. 5b, the cells were only transiently expressing empty vector, wild type, and mutant ADI1s as indicated.

### DNMT inhibition experiment

For inhibition of DNMT activity, the inhibitor, SGI-1027, was employed^46^. At 24 or 48 h after transfection, 2 μM of this compound was supplemented in culture medium for 16–24 h. The cells were then washed and collected for subsequent protein extraction or total RNA isolation.

### Analysis of DNA methylation status

The *CAV1* promoter was amplified as previously described^18^. The EpiTect Bisulfite kit (QIAGEN, 59104) was used to convert genomic DNA. The experiment was executed according to the standard protocol provided by the manufacturer. The amplicon was purified and then subjected into next-generation sequencing (NGS) and examined the methylated cytosine in *CAV1* promoter as described previously^47^. The methyl-seq analysis was employed for examining whole-genome methylation status and executed by subjecting 2 μg genomic DNA from either empty vector or ADI1-overexpressed J7 cells to TOOLS Co., Ltd. Analysis of sites with significant hyper- or hypo-methylation was conducted by using specific filters, such as the level of methylation either in Empty vector or in the ADI1 OE group could not be “0”, the difference of methylation level between these two groups should be more than 0.4 and the *q*-value (adjusted *p* value) should be smaller than 0.001. Only those genes with significant methylation status changes were candidates for further validation.

## Supplementary information

Supplementary Figure S1

Supplementary Figure S2

Supplementary Figure S3

Supplementary Figure S4

Supplementary Figure S5

Supplementary Figure S6

Supplementary Figure S7

Supplementary information

Supplementary Table S1

Supplementary Table S2

Supplementary Table S3
