# Supplementary figures and images for: The methionine salvage pathway-involving ADI1 inhibits hepatoma growth by epigenetically altering genes expression via elevating S-adenosylmethionine
Source: Cell Death Dis. 2019 Mar 11;10(3):240. doi: 10.1038/s41419-019-1486-4 (PMC6411897; doi:10.1038/s41419-019-1486-4)

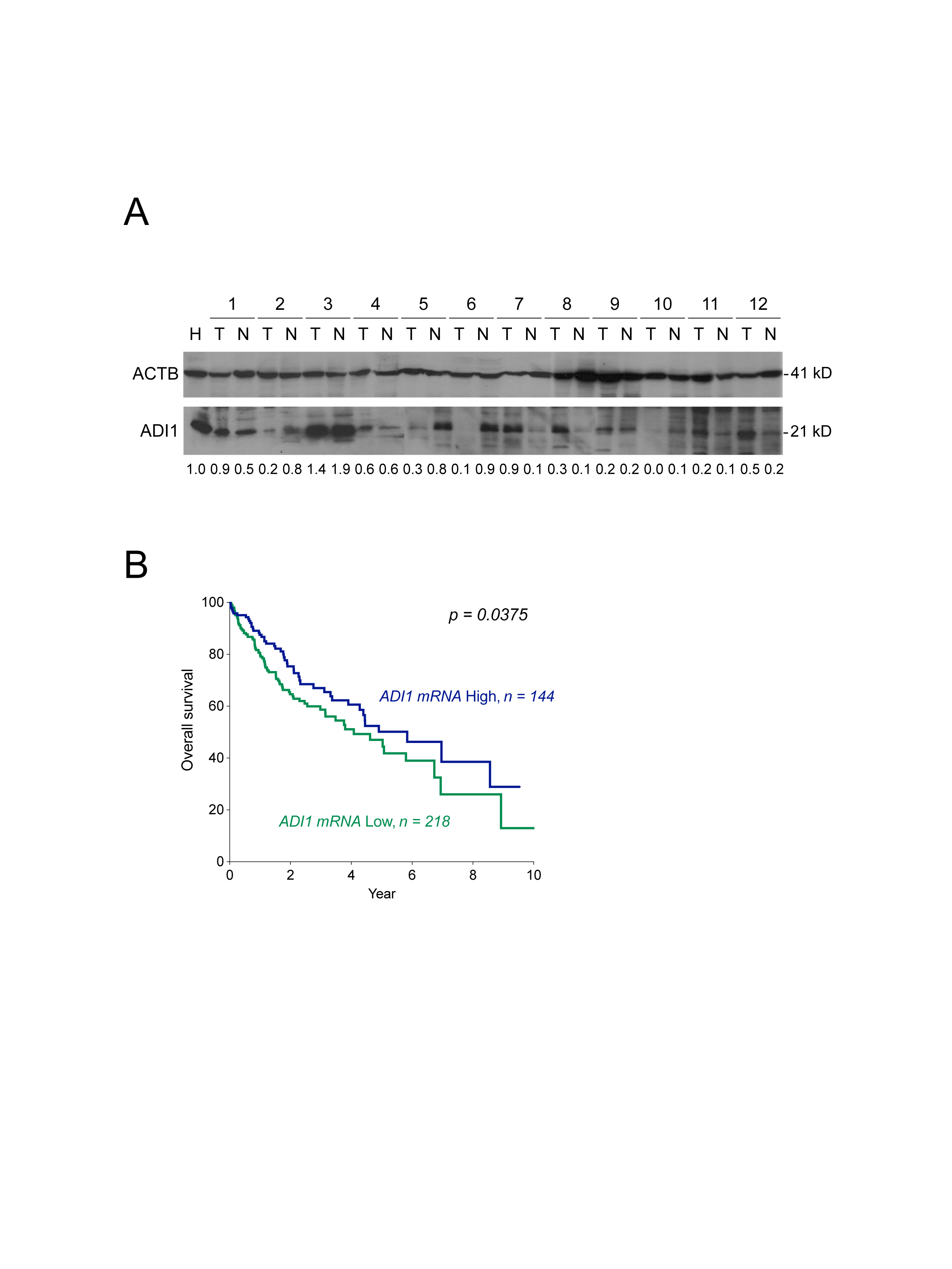

Supplement: Supplementary file 1 — Supplementary Figure S1 [file 41419_2019_1486_MOESM1_ESM.tif]

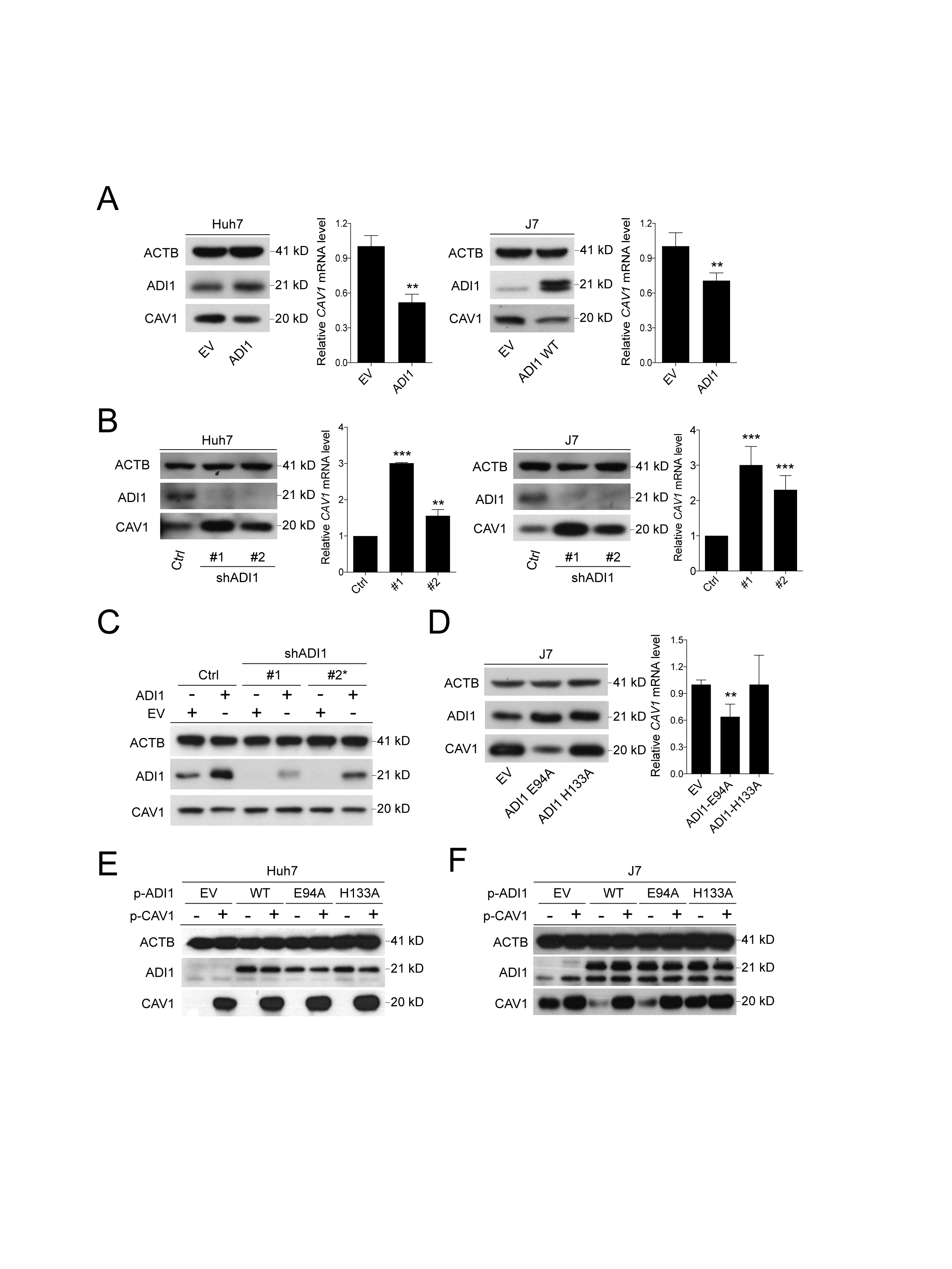

Supplement: Supplementary file 2 — Supplementary Figure S2 [file 41419_2019_1486_MOESM2_ESM.tif]

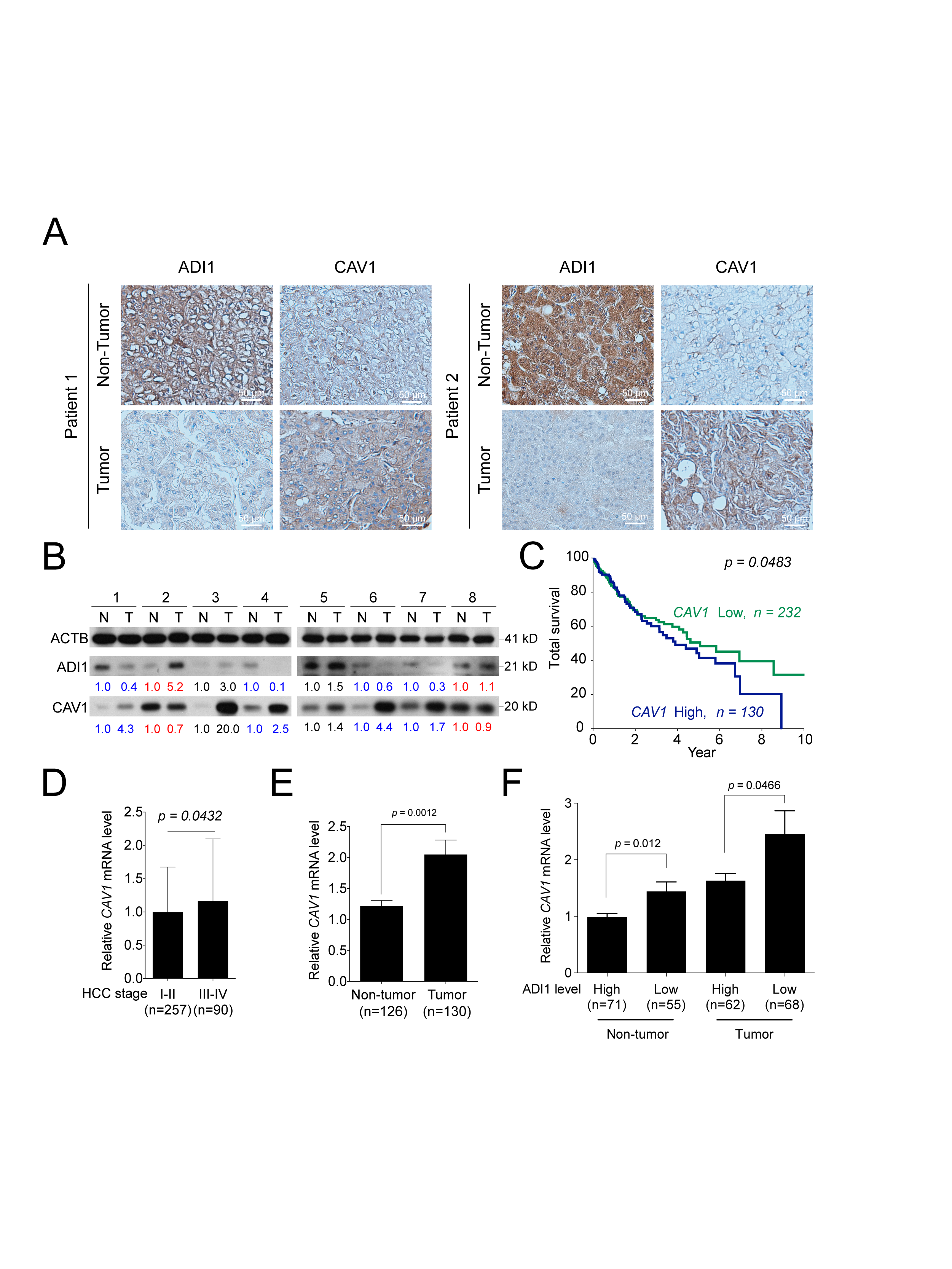

Supplement: Supplementary file 3 — Supplementary Figure S3 [file 41419_2019_1486_MOESM3_ESM.tif]

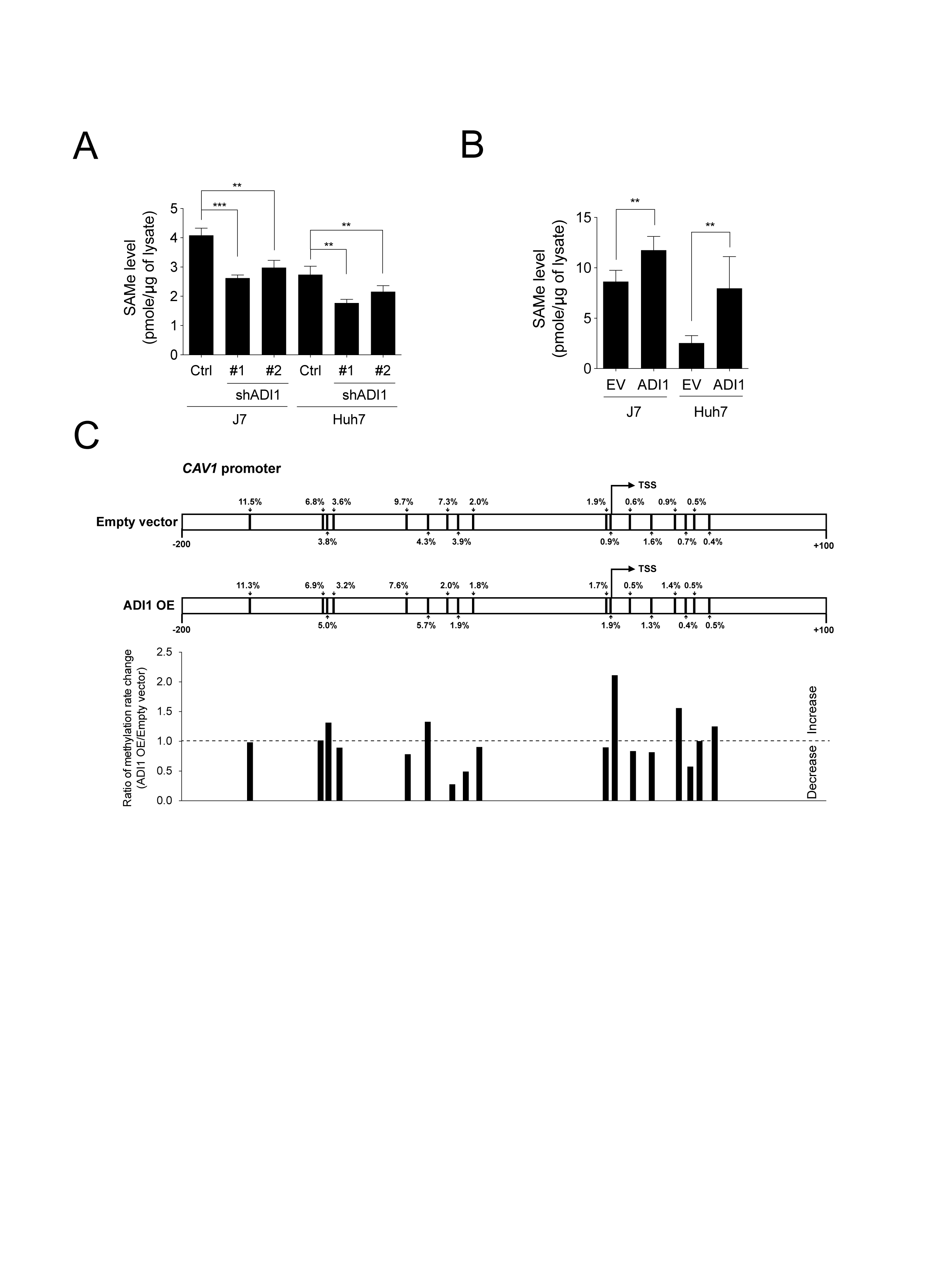

Supplement: Supplementary file 4 — Supplementary Figure S4 [file 41419_2019_1486_MOESM4_ESM.tif]

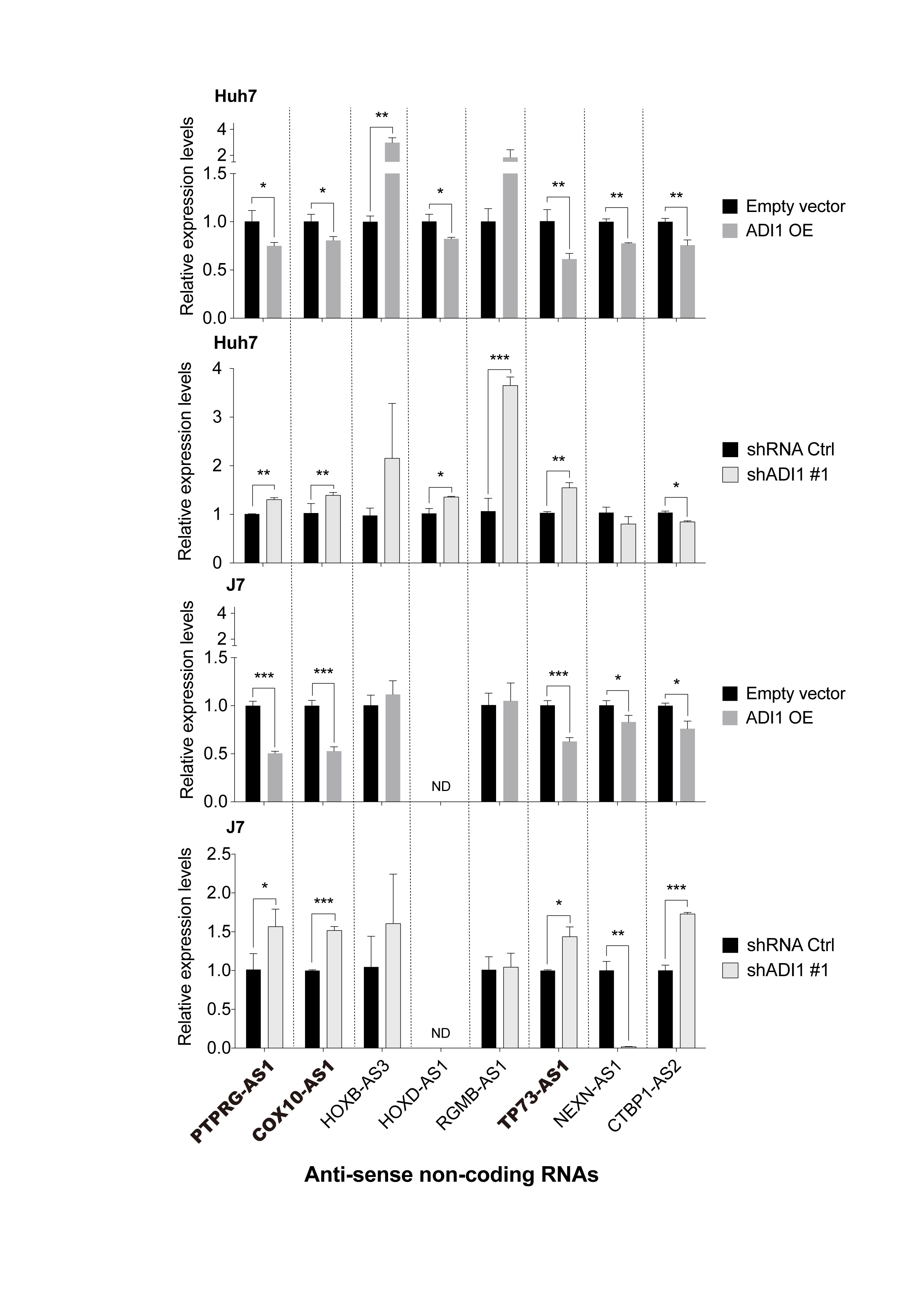

Supplement: Supplementary file 5 — Supplementary Figure S5 [file 41419_2019_1486_MOESM5_ESM.tif]

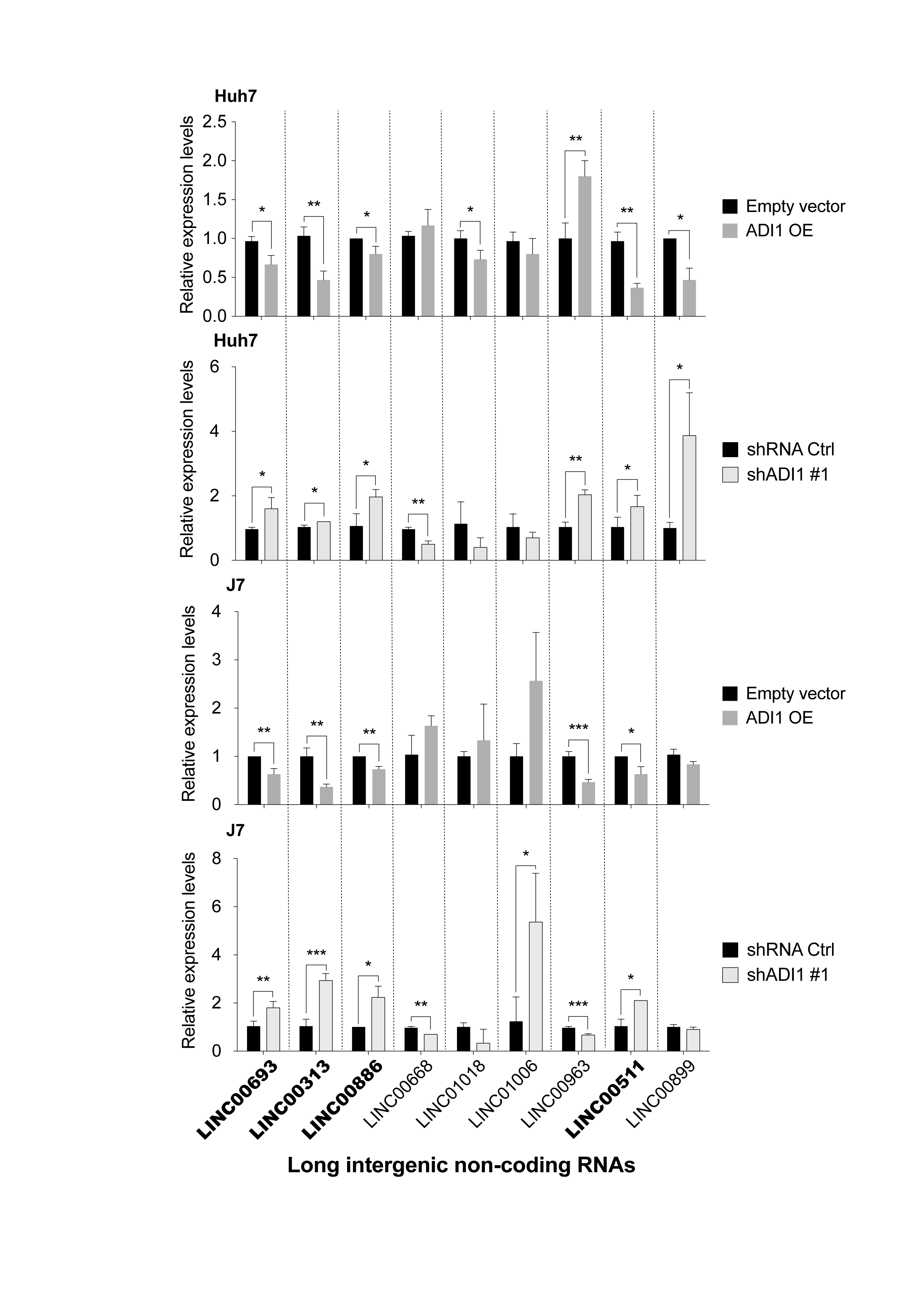

Supplement: Supplementary file 6 — Supplementary Figure S6 [file 41419_2019_1486_MOESM6_ESM.tif]

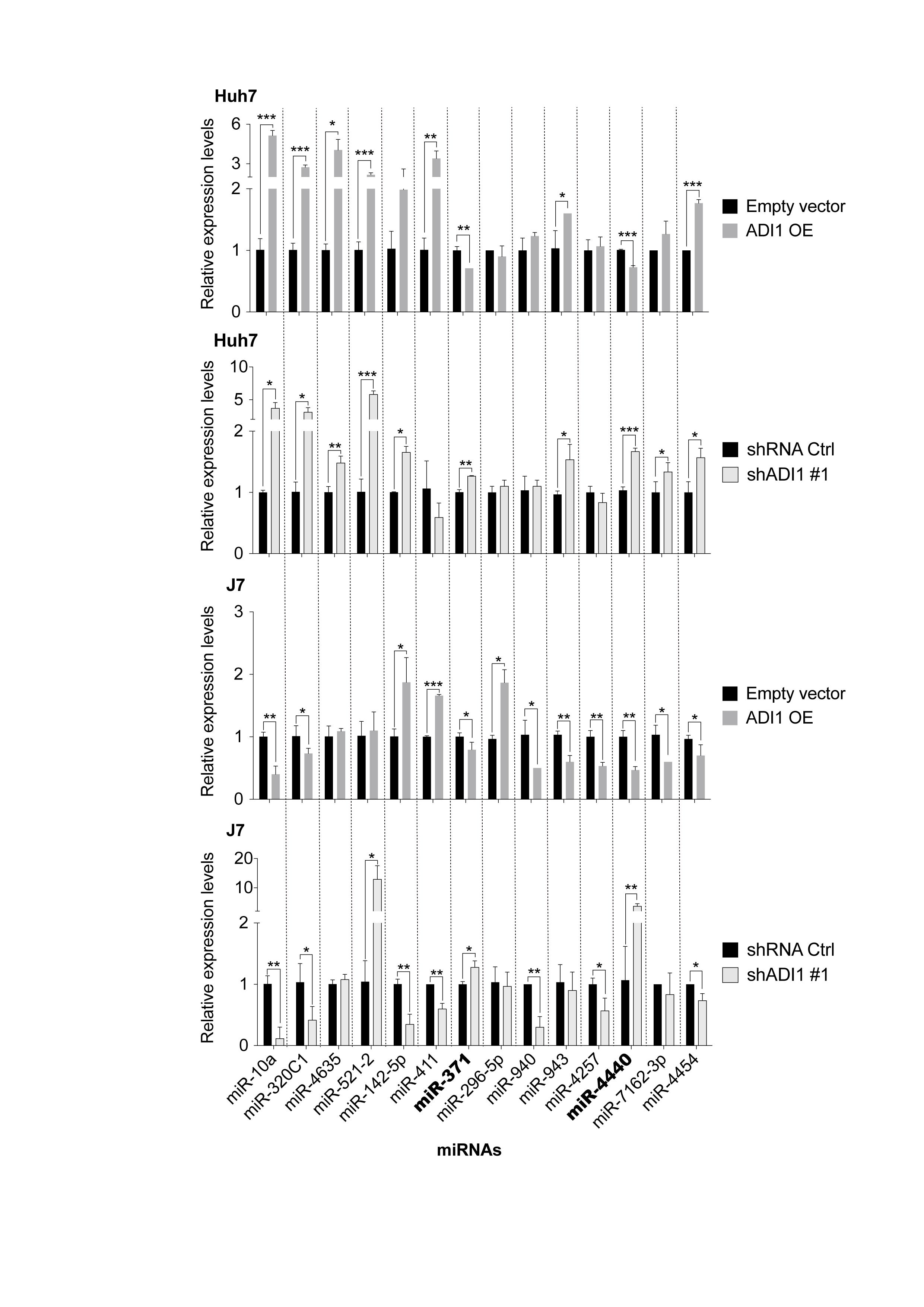

Supplement: Supplementary file 7 — Supplementary Figure S7 [file 41419_2019_1486_MOESM7_ESM.tif]
