# Supplementary material for: The methionine salvage pathway-involving ADI1 inhibits hepatoma growth by epigenetically altering genes expression via elevating S-adenosylmethionine
Source: Cell Death Dis. 2019 Mar 11;10(3):240. doi: 10.1038/s41419-019-1486-4 (PMC6411897; doi:10.1038/s41419-019-1486-4)
Supplement: Supplementary file 8 — Supplementary information [file 41419_2019_1486_MOESM8_ESM.docx]

**Supplementary information**

**Supplementary figure legends**

**Supplementary Figure S1.** (A TIFF file) The level of ADI transcript associated with prognosis of HCC patients. **(A)** The representative western blot analysis of lysates of paired tumorous and nontumorous tissues derived from HCC patients. H, the lysate of commercialized primary hepatocytes. Quantification of protein densitometries was performed by using imageJ software. **(B)** The TCGA data set was used to analysis the correlation between ADI1 mRNA level and HCC patients clinical outcome. Survival rate was analyzed by Kaplan-Meier analysis.

**Supplementary Figure S2.** (A TIFF file) ADI1 negatively modulates Caveolin-1 expression. Western blot analysis and RT-qPCR results of samples from **(A)** ADI1 overexpressed Huh7 and J7, **(B)** from ADI1 knocked-down Huh7 and J7 as well as **(C)** from ADI1 complemented J7 and **(D)** from mutated ADI1 overexpressed J7 cells. The *p* values were derived by using paired two-tail student *t*-test. * *p* < 0.05, ** *p* < 0.01, *** *p* < 0.001. Western blot analysis of **(E)** Huh7 or **(F)** J7 cells transfected by empty vector or ADI1-expressing plasmid with or without CAV1 overexpression. p-ADI1, ADI1 expressing plasmid. p-CAV1, CAV1 expressing plasmid.

**Supplementary Figure S3.** (A TIFF file) ADI1 was negatively correlated with CAV1 expression in HCC. **(A)** Representative IHC staining images of ADI1 and CAV1 in cancerous and non-cancerous tissues derived from HCC patients. **(B)** Representative western blot analysis of HCC patients-derived cancerous and noncancerous tissues. **(C)** The TCGA data set was used to analysis the correlation between CAV1 mRNA level and HCC patients clinical outcome. Survival rate was analyzed by Kaplan-Meier analysis. For cross-reference, the relative amounts of *CAV1* or *ADI1* transcripts were obtained from TCGA **(D)** or GSE14520 (**E and F**) dataset. *p* values were calculated by using unpaired two-tail student *t*-test.

**Supplementary Figure S4.** (A TIFF file) Alteration of ADI1 expression affects SAMe production and impacts on CAV1 promoter methylation status. The SAMe levels were detected in **(A)** ADI1 knocked-down and **(B)** overexpressed Huh7 and J7 cells. The *p* values were derived by using paired two-tail student *t*-test. * *p* < 0.05, ** *p* < 0.01, *** *p* < 0.001. **(C)** The Caveolin-1 promoter was amplified and then analyzed by NGS. The percentage of CpG island methylation at each site was as indicated. The ratio of percentage change was derived from ADI1 OE/Empty vector.

**Supplementary Figure S5.** (A TIFF file) Changing ADI1 expression impacts on anti-sense non-coding RNAs levels. The relative levels of indicated genes were detected by quantitative RT-PCR under ADI1 overexpression and knocked-down in either Huh7 or J7 cells. The *p* values were derived by using paired two-tail student *t*-test. * *p* < 0.05, ** *p* < 0.01, *** *p* < 0.001.

**Supplementary Figure S6.** (A TIFF file) Altering ADI1 levels effects on long intergenic non-coding RNAs (LincRNAs) expression. The relative levels of indicated lincRNAs were detected by quantitative RT-PCR under ADI1 overexpression and knocked-down in either Huh7 or J7 cells. The *p* values were derived by using paired two-tail student *t*-test. * *p* < 0.05, ** *p* < 0.01, *** *p* < 0.001.

**Supplementary Figure S7.** (A TIFF file) Variation in ADI1 levels orchestrates miRNAs expression. The relative levels of indicated miRNAs were detected by quantitative RT-PCR under ADI1 overexpression and knocked-down in either Huh7 or J7 cells. The *p* values were derived by using paired two-tail student *t*-test. * *p* < 0.05, ** *p* < 0.01, *** *p* < 0.001.

**Supplementary Table S1.** (A docx file) The baseline clinicopathological information of patients included in this study.

**Supplementary Table S2.** (A docx file) Primers used for detecting gene expression in this study.

**Supplementary Table S3.** (A xlsx file) The raw dataset of whole genome methyl-sequencing of cells with or without ADI1 overexpression.
