# Supplementary material for: The methionine salvage pathway-involving ADI1 inhibits hepatoma growth by epigenetically altering genes expression via elevating S-adenosylmethionine
Source: Cell Death Dis. 2019 Mar 11;10(3):240. doi: 10.1038/s41419-019-1486-4 (PMC6411897; doi:10.1038/s41419-019-1486-4)
Supplement: Supplementary file 9 — Supplementary Table S1 [file 41419_2019_1486_MOESM9_ESM.docx]

**Table S1.**

The baseline clinicopathological information of patients included in this study..

| Characteristics | Non-cirrhosis  (n = 83) | Cirrhosis  (n = 78) | P |
| --- | --- | --- | --- |
| Age, years | 53.8 ± 16.2 | 58.2 ± 13.1 | 0.057 |
| Gender, male, n (%) | 62 (74.7) | 57 (73.1) | 0.815 |
| Positive HBsAg, n (%) | 58 (69.9) | 53 (67.9) | 0.791 |
| Positive anti-HCV, n (%) | 13 (15.7) | 31 (39.7) | **0.001** |
| Alcoholism, n (%) | 22 (26.5) | 23 (29.9) | 0.636 |
| Ascites, n (%) | 6 (7.2) | 8 (10.3) | 0.496 |
| Tumor status |  |  |  |
| Microvascular invasion, n (%) | 28 (33.7) | 19 (24.4) | 0.191 |
| Macrovascular invasion, n (%) | 6 (7.2) | 7 (9.0) | 0.685 |
| Microsatellite, n (%) | 19 (22.9) | 9 (11.5) | 0.058 |
| Largest tumor size (cm) | 8.5 ± 5.0 | 4.9 ± 3.5 | **<0.001** |
| Tumor number >1, n (%) | 30 (36.1) | 26 (33.3) | 0.708 |
| Capsule, n (%) | 60 (72.3) | 59 (75.6) | 0.628 |
| Histology grade ≧3, n(%) | 55 (66.3) | 41 (52.6) | 0.077 |
| Biochemistry |  |  |  |
| Bilirubin (mg/dL) | 1.4 ± 2.1 | 1.2 ± 1.0 | 0.449 |
| AST (U/L) | 109 ± 149 | 72 ± 55 | **0.039** |
| ALT (U/L) | 90 ± 122 | 64 ± 56 | 0.090 |
| Albumin (g/dL) | 3.8 ± 0.7 | 3.7 ± 0.5 | 0.398 |
| Creatinine (mg/dL) | 1.3 ± 1.7 | 1.1 ± 0.6 | 0.331 |
| Prothrombin time (sec) | 12.2 ± 1.5 | 12.6 ± 1.4 | 0.066 |
| Alpha-fetoprotein (ng/mL) | 25 (2.9 – 327500) | 30 (1.5 – 89637) | 0.309 |
