# Supplementary material for: The methionine salvage pathway-involving ADI1 inhibits hepatoma growth by epigenetically altering genes expression via elevating S-adenosylmethionine
Source: Cell Death Dis. 2019 Mar 11;10(3):240. doi: 10.1038/s41419-019-1486-4 (PMC6411897; doi:10.1038/s41419-019-1486-4)
Supplement: Supplementary file 10 — Supplementary Table S2 [file 41419_2019_1486_MOESM10_ESM.docx]

**Table S2. Primers used for detection of gene expression in this study.**

| Primer names | Sequences |
| --- | --- |
| GAPDH qPCR_F | AATGAAGGGGTCATTGATGG |
| GAPDH qPCR_R | AAGGTGAAGGTCGGAGTCAA |
| CAV1 qPCR_F | CAGGCTTGTAACCTTTACAGGAC |
| CAV1 qPCR_R | CA T AGA TGCTT AGTCCCTCA TGC |
| PTPRG-AS1 F | ACTGA AGAGGA ACCAGGGA CAG |
| PTPRG-AS1 R | GGGGAAACTGAAATGAATG A AG |
| COX10-AS1 F | AACCGCAAAGCACGGTGTG |
| COX10-AS1 R | TGC CAT ACT CAC ACC GGT CA |
| HOXB-AS3 F | AATCCCATTTCCTAGCCTGG |
| HOXB-AS3 R | ACCTCCATCAATTCCATGAG |
| HOXD-AS1 F | ACCTGCCTCTACTACTGCAAA |
| HOXD-AS1 R | GCAAAGACAATATAAGGGCCC |
| RGMB-AS1 F | AGTGGGCAAACTTCAACGTTC |
| RGMB-AS1 R | GAGCTGCCATTGAATTAATCCG |
| TP73-AS1 F | CCGGTTTTCCAGTTCT TGCAC |
| TP73-AS1 R | GCCTCACAGGGAAACTTCATGC |
| NEXN-AS1 F | GAATAAAGATGGATTCTCGCTC |
| NEXN-AS1 R | AATTGCTTGAACCTGGGAG |
| CTBP1-AS2 F | TTACTGCAACCTCTGCCTC |
| CTBP1-AS2 R | ATCTAAAGATCTGTAGTCCCAGG |
| LINC00693 F | GAAATCACTTCAAGCCTCCA |
| LINC00693 R | GTAGTCCATGGCATCTCTG |
| LINC00313 F | AGTTGCTTCCTCTGGAGTC |
| LINC00313 R | TCATTTCCTTCCCAGGGAC |
| LINC00886 F | CTCATTTGGAAGCAGTGCA |
| LINC00886 R | CATGATGTGACCATGACTCTC |
| LINC00668 F | CTGAAGCAGACAGGATCTC |
| LINC00668 R | GAGAATTGCTTGAGGGAGAG |
| LINC01018 F | GTAAATGAGGCTCCTGGGA |
| LINC01018 R | ATATTCTGTCTCCACCGCC |
| LINC00982 F | GAAGAGCTGAACCAATGGG |
| LINC00982 R | CTGCTTCTGCCTCTAAGGA |
| LINC01006 F | GGTCTCATGGAAATGGCAG |
| LINC01006 R | AATGCGTGAAGAAAGGCTG |
| LINC00963 F | GTACAGTTCCTCCCTCAGG |
| LINC00963 R | CACCACAGCAGCTAATGTC |
| LINC00511 F | AGAACGTGGTGGAATCAGAG |
| LINC00511 R | CTTCACTGCAGATTCGACG |
| LINC00899 F | CTGAGGGTACTGTCCACTC |
| LINC00899 R | CAATTATGTAACTCTCAGAGCCTC |
| Universal RT | AAC GAG ACG ACG ACA GAC TTT TTT TTT TTT TTT VN |
| Universal reverse | AAC GAG ACG ACG ACA GAC TTT |
| miR-10a qPCR F | CCT GTA GAT CCG AAT TTG TGA AA |
| miR-320C1 qPCR F | AGCTGGGTTGAGAGGGTAAA |
| miR4635 qPCR F | TGAAGTCAGAACCCGCAAAAA |
| miR-521-2 qPCR F | GCACTTCCCTTTAGAGTGTAAA |
| miR-142-5p qPCR F | TAAAGTAGAAAGCACTACTAAA |
| miR-411 qPCR F | AGT AGA CCG TAT AGC GTA CGA AA |
| miR371B qPCR F | CAAAAGATGGCGGCACTTTAAA |
| miR296-5p qPCR F | CCCCCCCTCAATCCTGTAAA |
| miR940 qPCR F | CAGGGCCCCCGCTCCCCAAA |
| miR943 qPCR F | ACTGTTGCCGTCCTCCAGAAA |
| miR-4257 qPCR F | CAG AGG TGG GGA CTG AGA AA |
| miR-4440 qPCR F | TGGGGCTTGCTGGCTTGAAA |
| miR-7162-3p qPCR F | TCTGAGGTGGAACAGCAGCAAA |
| miR-4454 qPCR F | TCCGAGTCACGGCACCAAAA |
